# Tuberculose hépatique nodulaire: complication inhabituelle au cours de la maladie de Wilson

**DOI:** 10.11604/pamj.2014.17.22.2380

**Published:** 2014-01-17

**Authors:** Ali Zinebi, Adil Rkiouak, Youssef Akhouad, Ahmed Reggad, Zohor Kasmy, Mostapha Boudlal, Monsef Rabhi, Khalid Ennibi, Jilali Chaari

**Affiliations:** 1 Service de Médecine A, Hôpital Militaire d’ Instruction Mohamed V, Rabat, Maroc

**Keywords:** Maladie de Wilson, tuberculose, foie, Wilson disease, tuberculosis, liver

## Abstract

La tuberculose hépatique nodulaire est rare. Nous rapportons une forme pseudo-tumorale dont le tableau clinico-biologique et radiologique initial était aspécifique. Il s'agit d'un jeune marocain suivi pour maladie de Wilson et présentant une fièvre au long cours. L'imagerie met en évidence une lésion nodulaire hépatique non spécifique. L'examen anatomo-pathologique au cours d'une biopsie écho guidée du nodule hépatique permit de porter le diagnostic. L’évolution clinique est favorable sous traitement spécifique.

## Introduction

L'apparition d'un nodule au sein d'un foie d'hépatopathie chronique fait évoquer et redouter l'hypothèse d'un carcinome hépatocellulaire (CHC). Le cas clinique que nous rapportons n'est pas conforme à ce schéma habituel puisque l'enquête étiologique a retrouvé une cause inattendue au regard du contexte clinique, et heureusement de meilleur pronostic.

## Patient et observation

Un jeune marocain de 16 ans, vacciné au BCG, est suivi depuis un an pour une maladie de Wilson. Le diagnostic a été retenu lors d'un bilan de cytolyse chronique avec un anneau cornéen de Keyser Fleischer et un syndrome extrapyramidal. L'IRM cérébrale montrait des hyper signaux thalamo-mésencéphaliques. L’étude génétique confortait le diagnostic en retrouvant la mutation homozygote C2752G >Ap. Asp 918 Asn. Le patient est depuis mis sous D penicillamine à raison de 900 mg/j. L’évolution a été jugée favorable devant l'amélioration de la fonction hépatique avec la disparition de l'anneau de Keyser Fleischer et du syndrome extrapyramidal.

Un mois avant son admission dans notre formation, le patient présentait un syndrome fébrile avec sueur profuse surtout la nuit évoluant dans un contexte d'amaigrissement chiffré à 6 kg.

L'examen clinique ne retrouvait pas de foyer infectieux évident. Le bilan biologique montrait un syndrome inflammatoire avec une protéine C réactive à 50mg/l sans hyperleucocytose, une détérioration de la fonction hépatique jusque là stable avec des ALAT (Alanine amino transférase) à 120 UI/l (VN: 15- 35UI/l), des ASAT (Aspartate amino transférase) à 95 UI/l ( VN: 20- 45UI/l), des GGT (Gamma glutamyl transférase) à 170 UI/l (VN: 25- 50 UI/l), PAL ( Phosphatases alcalines) à 140 UI/l ( VN: 85- 160 UI/l) et une bilirubinémie normale. Le taux de prothrombine était à 30%. L'alpha foeto protéine à 150 ng/ml (VN: <10ng/ml). La procalcitonine était normale, Les hémocultures, l'examen cytobactériologique des urines, la recherche de bacille de Kock aux tubages gastriques et aux urines était négative. L'intradermoréaction à la tuberculine était négative. Les sérologies virales B, C et VIH, les sérologies hydatiques et amibiennes ainsi que le sérodiagnostic de Widal et Félix et la sérologie de Wright étaient négatives.

**Figure 1:**
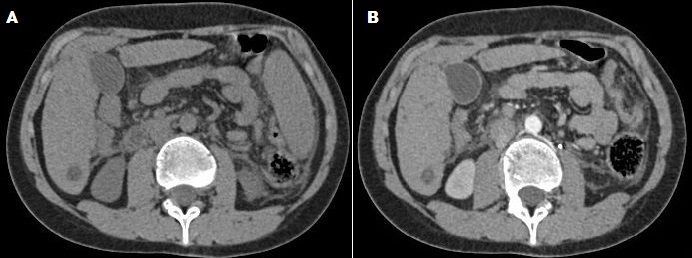
A) TDM abdominale avant injection de produit de contraste en coupe axiale montrant un nodule hépatique hypodense du segment VI de 15 mm de diamétre; B)TDM abdominale après injection de produit de contraste en coupe axiale montrant une discrète prise de contraste périphérique en couronne du nodule hépatique du segment VI.

**Figure 2:**
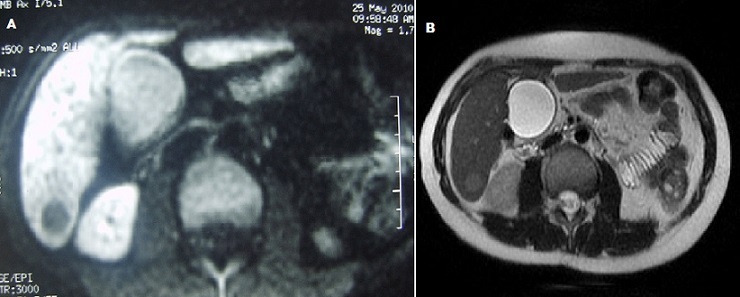
A) Coupe axiale en diffusion B500 montrant un nodule hépatique en hyposignal diffusion; B)Coupe axiale en pondération T2 montrant un nodule hépatique du segment VI en discret hypersignal par rapport au foie.

L’échographie hépatobiliaire montrait une image hypoéchogène du segment V. Le scanner abdominale montrait un nodule hépatique du segment VI de 15 mm de diamètre prenant discrètement le contraste en couronne périphérique (Figure 1). L’‘IRM hépatique objectivaient de multiples lésions nodulaires hépatiques de taille infra centimétriques et un macro nodule de 15 mm au niveau du segment VI. Ces nodules sont hypo intenses sur les séquences T1 et sur les séquences de diffusion. Ils sont légèrement hyper intenses sur les séquences T2 (Figure 2). Il n'existait ni ascite ni adénopathies profondes.

**Figure 3:**
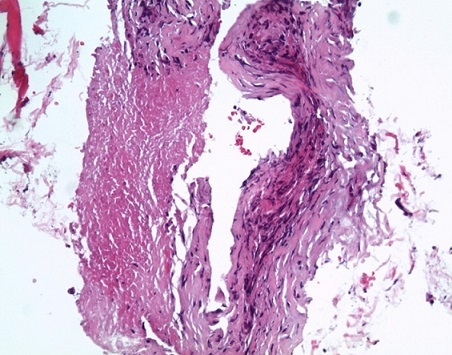
Biopsie du nodule hépatique: Tissu fibreux et inflammatoire bordant une plage de nécrose caséeuse. Les éléments inflammatoires au sein de ce tissu fibreux sont constitués de cellules histiocytaires épithélioïdes mêlées à des lymphocytes et ébauchant un granulome.

La biopsie hépatique écho guidée dirigée sur le macro nodule retrouvait un aspect de granulome épithélio-gigonto-cellulaire avec nécrose caséeuse (Figure 3). L’étude bactériologique montrait la présence de bacille alcoolo acido résistant (BAAR).

Le patient était mis sous anti bacillaires associant Isoniazide 5mg/kg, Rifampicine 10 mg/kg, Ethambutol 25 mg/kg et Pyrazinamide 30 mg/kg pendant deux mois et Isoniazide avec Rifampicine pendant sept mois. Après 9 mois total de traitement, L’évolution clinique et biologique était favorable avec apyrexie, prise de poids et disparition du syndrome inflammatoire. La fonction hépatique s'est améliorée avec un TP à 82% à l'arrêt du traitement. L'IRM hépatique de contrôle à l'arrêt du traitement ne retrouvait plus de nodule.

## Discussion

La maladie de Wilson est une maladie génétique autosomique récessive due à une anomalie du gène ATP7β du chromosome 13. Les lésions hépatiques nodulaires sont rares au cours de la maladie de Wilson notamment chez l'enfant. Les principales lésions hépatiques chez l'enfant sont l'hépatoblastome, l'hémangiome, l'hémangio-endoththelium, le lymphome, l'hématome, la sclérose tubéreuse associé à de multiple tumeur hépatique lipomateuse et l'hyperplasie nodulaire régénérative [1].

La maladie de Wilson peut se compliquer de tumeurs malignes (TM) hépatiques. Dans la littérature, seul une vingtaine de TM primitive ont été rapportées [2]. Les mécanismes incriminés sont: l'accumulation de cuivre en intracellulaire entraînant une nécrose des hépatocytes associée à une prolifération des canaux biliaires [3], l'effet du traitement chélateur du cuivre est bien connu. En effet en diminuant les taux tissulaires de cuivre, il pourrait favoriser la survenue d'un hépato carcinome [3]. L'autre mécanisme pouvant expliquer la survenue de tumeur maligne hépatique serait un excès de fer intrahépatocytaire [3].

L′atteinte hépatique, fréquente au cours de la maladie tuberculeuse, s′intègre habituellement dans un tableau de tuberculose disséminé. La tuberculose hépatique autonome est rare même dans un pays à forte endémicité tuberculeuse comme le maroc. Elle se définit par l′absence de splénomégalie, d′entérite tuberculeuse, et d′image miliaire à la radiographie pulmonaire. L'atteinte hépatique de la tuberculose peut être classée en tuberculose miliaire, tuberculose pulmonaire avec atteinte hépatique, tuberculose primitive du foie, tuberculoses focales ou abcédés et la cholangite tuberculeuse. La forme la plus commune de l′atteinte hépatique est la forme miliaire de la tuberculose retrouvée dans 80% des cas. Le mécanisme étant une dissémination hématogène à travers l′artère hépatique [4]. La localisation focale tuberculeuse pseudo tumorale du foie est rare [5] en raison de la teneur faible en oxygène dans le foie, ce qui est défavorable à la croissance des mycobactéries [6, 4]. L'atteinte hépatique nodulaire chez notre patient est certainement due à un état d'immunodépression engendré par le traitement immunosuppresseur de la maladie de Wilson ou encore une confluence de micronodules d'autant plus que l'IRM hépatique montrait d'autres micronodules hépatiques. En effet, les localisations extra pulmonaires sont fréquentes chez les sujets immunodéprimés notamment VIH positifs.

La tuberculose hépatique peut se présenter dans un tableau d'hypertension portale. [7]

Les aspects radiologiques ne sont pas spécifiques pouvant faire évoquer une lésion maligne comme c'est le cas dans notre observation d'autant plus que l'alpha foeto protéine était élevé. Elle se manifeste en échographie sous forme d'une ou plusieurs masses hypoéchogène avec ou sans renforcement postérieur [8], renfermant parfois des calcifications. En TDM, l'aspect dépend du stade évolutif de la maladie. Les lésions au début sont isodenses. Elles deviennent hypodenses par nécrose caséeuse puis finissent par se calcifier. L'injection de produit de contraste entraîne un rehaussement annulaire. Lorsque la caséification est importante au centre du granulome tuberculeux, elle entraîne la formation d'abcès tuberculeux. Ils se présentent en TDM sous forme de lésions kystiques à contours polylobés, non rehaussées ou rehaussées de façon modérée en périphérie [9].

En IRM, l'aspect des lésions est variable. L'aspect le plus évocateur est celui d'une lésion hyperintense en périphérie, de moindre intensité au centre, sur les séquences pondérées T2, et qui se rehausse en périphérie après injection de contraste. Les lésions kystiques sont en hyposignal T1, et en hyper T2 [9].

La recherche du bacille de Koch à l'examen direct et à la culture est inconstamment positive.

L'histologie hépatique notamment par ponction biopsie écho-guidée ou scan guidée reste un moyen diagnostique important. Elle permet la mise en évidence de matériel nécrotique, caséeux, associé dans 25% des cas à un granulome épithélio-gigantocellulaire [6].

## Conclusion

Une tuberculose hépatique isolée, quoique rare, doit faire partie du diagnostic différentiel devant une masse survenant sur un foie d'hépatopathie chronique. En zone d'endémie tuberculeuse. La ponction biopsie reste nécessaire en cas de doute notamment si les critères diagnostiques du carcinome hépatocellulaire ne sont pas réunis.
